# Electrocardiography Interpretation Proficiency Among Medical Doctors of Different Grades in the United Kingdom

**DOI:** 10.7759/cureus.29755

**Published:** 2022-09-29

**Authors:** Ahmed Ali Abdalla, Dibbendhu Khanra

**Affiliations:** 1 Internal Medicine, Manchester University NHS Foundation Trust, Manchester, GBR; 2 Cardiology, Liverpool Heart and Chest Hospital, Liverpool, GBR

**Keywords:** clinical competence, arrhythmia, medical education, interpretation, electrocardiography

## Abstract

Objective: Electrocardiography interpretation is a core clinical skill for all doctors participating in emergency medical services. Given the majority of ECGs initially performed in hospital on admission are reviewed by junior doctors, and the need for life-threatening pathology to be diagnosed or excluded, further understanding of the level of competency in interpretation and factors associated with this are needed.

Methods: This was a cross-sectional, descriptive, analytical study. The data were collected using a structured questionnaire. This was comprised of two sections; the first section contained questions related to confidence, previous ECG learning and factors thought to be associated with ECG interpretation competence. The second section was an ECG quiz of 10 12-lead ECGs of varying complexity for interpretation assessment. Descriptive and inferential statistics were utilized for data analysis.

Results: Sixty-two doctors from foundation year 1 to registrar level working in acute medicine across three hospitals participated. The mean overall percentage score for the ECG quiz was 45%. No association was found between junior doctor training grade and overall score on the ECG assessment. Undergraduate and postgraduate teaching strategies also did not impact competence. Only 9.7% reported themselves as “confident” interpreting ECGs. There was a trend towards higher levels of competency among those who felt they had undergone sufficient ECG teaching and those who sought regular feedback from other clinicians.

Conclusion: This study demonstrated low overall levels of ECG interpretation competency among junior doctors in a large acute teaching NHS trust regardless of grade. Factors associated with competency remain unclear.

## Introduction

Electrocardiography (ECG) remains a simple, effective, and widely used investigation for rapid diagnosis in the acute clinical setting [1]. ECG interpretation is a core clinical skill for all doctors participating in emergency medical services in the United Kingdom (UK) and around the world. A multitude of studies since the 1990s has demonstrated significant variability in interpretation skills among medical students and medical practitioners, some showing concerningly poor levels of proficiency [2,3]. These studies have led to attempts at improving ECG teaching and learning at both the undergraduate and postgraduate levels [4]; however, there remains no standardized optimal method of improving ECG interpretation skills among the various level of trainees. In addition to the scarcity of optimal teaching methods, there is a paucity in the literature on the factors that impact level of proficiency in interpretation.

Accurate and timely interpretation of the ECG abnormalities of many medical presentations is paramount in the acute setting [1]. Given the majority of ECGs performed in hospital on admission are reviewed by junior doctors and the need for potentially serious abnormalities to be diagnosed or excluded, further understanding of level of competency in interpretation and the factors associated with this are needed.

The lack of literature in this regard impedes the development of more robust targeted training methods for junior doctors in the National Health Service (NHS). Considering this, the authors aimed to assess the level of proficiency of junior doctors at ECG interpretation, their confidence, and potential factors associated with competency.

This article was previously presented as a meeting abstract at the American College of Cardiology Conference 2022 on March 8, 2022.

## Materials and methods

This was a cross-sectional, descriptive, analytical study carried out between April and May 2021. Eligible participants for the study were junior doctors (foundation year 1 to specialist registrar level) contributing to the acute medical on-call rota at the time of the study in Manchester University Hospitals NHS Foundation Trust, namely three hospitals, Manchester Royal Infirmary, Wythenshawe Hospital, and Trafford General Hospital. Doctors in training and non-training posts were invited to participate. Participation was entirely voluntary. Consultants and specialty and associate ataff (SAS) doctors were not invited to take part.

Sampling was non-probability and via full coverage of all junior doctors in the above-specified capacity. The data were collected using a structured questionnaire sent to participants as a Google forms link to each hospital’s general medical on-call Whatsapp group accompanied by a message outlining the purpose of the study and inclusion criteria. In addition, this was also disseminated individually to eligible doctors’ registered professional emails requesting them to participate. The Google form was available for one month from the time of sending, could only be completed once and incentive for participating was enhanced by sending the answers to those who requested.

The questionnaire was comprised of two sections; the first section contained questions related to factors thought to be associated with ECG interpretation competence based on literature review, which are listed as independent variables below. The second section was an ECG quiz of 10 12-lead ECGs of varying complexity for the junior doctor to interpret. Each question began with a brief history in the form of the patient’s main complaint. Answering was by typing in free text diagnoses such as “anterolateral ST-elevation myocardial infarction (STEMI)” or “atrial flutter with 2:1 block”. Guidance on this was provided within the questionnaire prior to the participant completing the second section.

The 10 ECGs included in the assessment were as follows: (1) atrial fibrillation, (2) complete heart block, (3) atrial flutter with 2:1 block, (4) anterolateral STEMI, (5) large pericardial effusion/cardiac tamponade (illustrated by electrical alternans in conjunction with history given), (6) severe hyperkalemia, (7) posterior MI, (8) pre-excited atrial fibrillation, (9) dual chamber pacing, and (10) tri-fascicular block.

The quiz was written by the author (AA) and reviewed by a general medical consultant and a cardiologist (DK) for validity, accuracy, and relevance to the acute medical take. Answers to each question were reviewed individually and allocated scores of 0, 0.5 (if the answer was incomplete), or 1 as agreed between the three contributors to the quiz. Overall scores for the quiz were then calculated and each participant was given a score out of 10 for the assessment.

Variables of the study

The independent variables of the study included undergraduate medical training whether in the UK or overseas, form of undergraduate and postgraduate ECG training, previous independent ECG training/study, current level of medical training, years since graduation, approximate number of ECGs usually interpreted per week, previous cardiology rotation or not, whether the doctor thought they had sufficient ECG training, frequency of checking ECG interpretation. The dependent variables were self-perceived confidence and level of proficiency based on the overall score on ECG quiz.

Descriptive and inferential statistics were used for data analysis and the software used was Statistical Package for the Social Sciences (SPSS version 27; Armonk, NY: IBM Corp.). Statistical tests of association used were Kruskal-Wallis H (non-ordered categories) and Jonckherre-Terpstra (ordered categories) tests.

The study was approved by the Department of Research and Innovation at Manchester University NHS Foundation trust. Informed written consent was obtained from all participants and responses to the questionnaire were completely anonymized. The authors declare no conflicts of interest.

Patient and public involvement

This study did not use any patient information. Patients and the public were not involved in the development or conduct of this study. All electrocardiograms used in the study were anonymized and free of any patient identifiable information.

## Results

Sixty-two junior doctors responded to the invitation to participate of approximately 230 doctors on the acute medical on-call rota at the three hospitals in the study, giving a response rate of 27%.

Junior doctor characteristics

There was a variety of grades of doctors participating with foundation year 1 doctors (FY1), senior house officers (this term is no longer in use but is mentioned here for simplicity as it encompasses all of the following on the acute medical rota FY2s, junior clinical fellows, core medical trainees, GP trainees) and registrars accounting for 27.4%, 45.2%, and 27.4% of the study population respectively. A total of 67.7% of participants graduated from the UK while 32.3% graduated from non-UK medical schools. In terms of years of experience, approximately 40% of junior doctors had six years or more of experience since graduation; this is detailed further in Table 1 showing trainee characteristics.

**Table 1:** Junior doctor characteristics

Question	Response	n (%)
Location of undergraduate medical training	UK	42 (67.7%)
	Overseas	20 (32.3%)
Years since graduation	0-2	15 (24.2%)
	3-5	22 (35.5%)
	6-10	14 (22.6%)
	More than 10	11 (17.7%)
Medical grade	FY1	17 (27.4%)
	SHO	28 (45.2%)
	Registrar	17 (27.4%)

Types of ECG teaching

**Figure 1:**
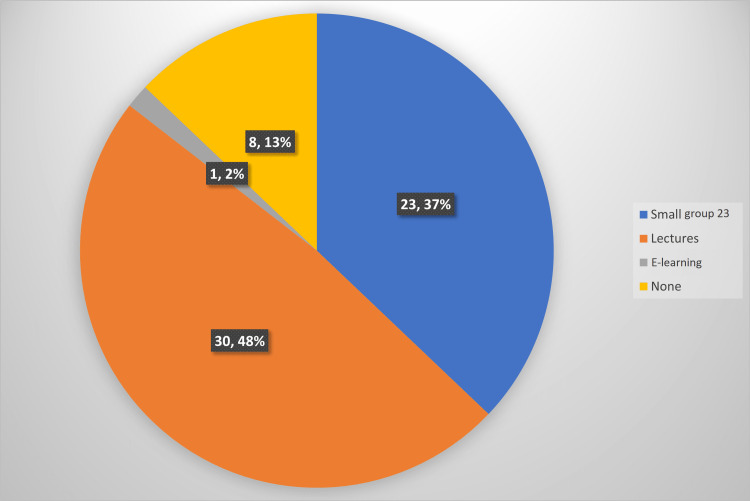
Type of undergraduate ECG teaching undertaken

**Figure 2:**
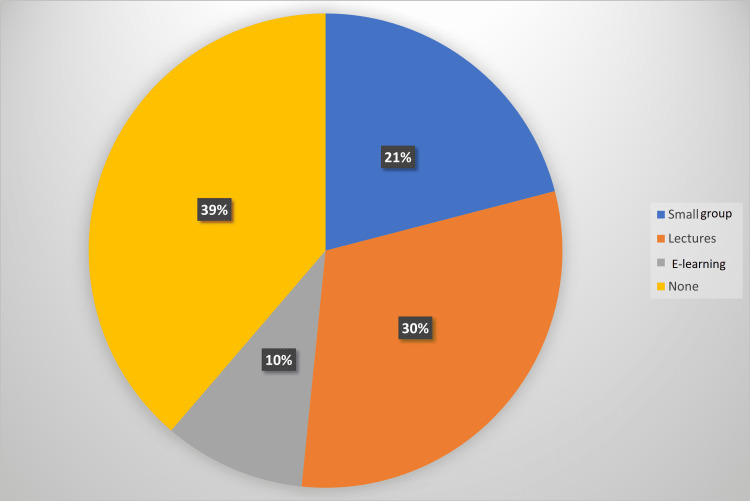
Type of postgraduate ECG teaching undertaken

Of 62, 30 (48.4%) participants had traditional lectures as their main ECG teaching during undergraduate training, and 37.1% said that small-group tutorials were the predominant ECG teaching format at their medical school (Figure 1). With regard to postgraduate ECG teaching, 38.7% of junior doctors did not receive any (Figure 2). Online-based modules were more prevalent in postgraduate teaching (9.7%) compared to undergraduate teaching (1.6%) as reported in our study population.

**Figure 3:**
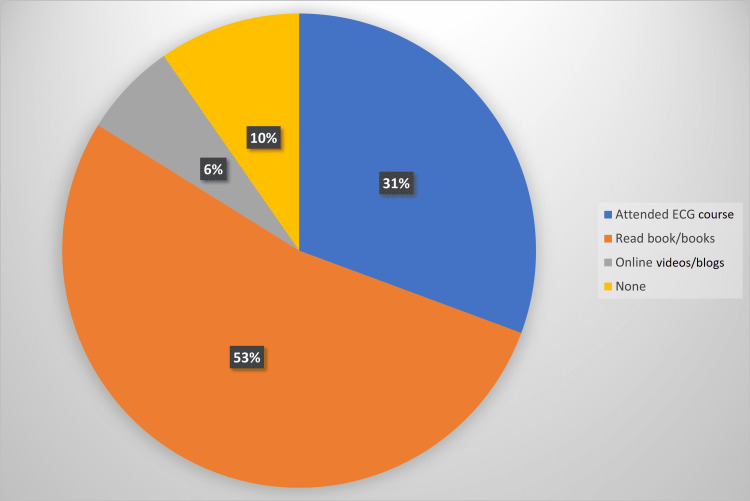
Independent ECG learning undertaken

The majority (53.2%) of participants read an ECG book as independent learning, followed by use of online resources such as videos or blogs (30.6%), 6.5% attended an ECG interpretation course and 9.7% undertook no further ECG learning outside of undergraduate and postgraduate formal teaching (Figure 3).

Confidence

**Figure 4:**
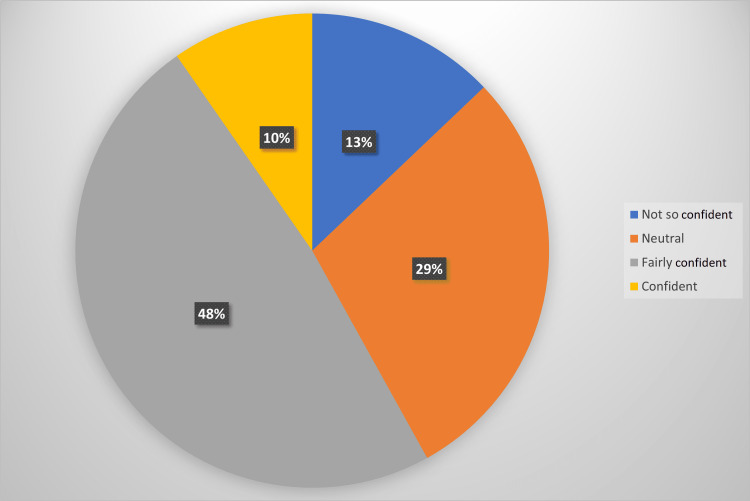
Confidence in ECG interpretation among doctors

Only 26 (41.9%) of the 62 participants felt they generally had sufficient ECG training. Confidence was assessed using an adapted four-point Likert scale. Further, 9.7% reported themselves as “confident” interpreting ECGs while 41.9% were either neutral or not so confident. The most frequent response was “fairly confident” (48.4% of trainees) as illustrated in Figure 4.

ECG quiz scores and associated factors

The mean overall score percentage for the ECG quiz was 45% (4.5 out of 10), participants were more familiar with common ECG abnormalities and struggled to identify ECG patterns for less common diagnoses, particularly Atrial fibrillation with Wolff-Parkinson-White syndrome or pre-excited atrial fibrillation and tri-fascicular block, less than 20% of doctors interpreting these correctly. The vast majority of trainees (74.2%) also misinterpreted the ECG pattern of a posterior myocardial infarction; this was frequently labeled as “NSTEMI” or “myocardial ischemia”. Table 2 illustrates the distribution of scores for each question.

**Table 2:** Distribution of scores to individual questions in the ECG assessment STEMI: ST-elevation myocardial infarction

Question	Score of 0 points (incorrect)	Score of 0.5 points (incomplete answer)	Score of full 1 point (correct and complete)
Q1. Atrial fibrillation	13 (21%)	0	49 (79%)
Q2. Complete heart block	26 (41.9%)	0	36 (58.1%)
Q3. Atrial flutter with 2:1	30 (48.4%)	0	32 (51.6%)
Q4. Anterolateral STEMI	0	26 (41.9%)	36 (58.1%)
Q5. Cardiac tamponade	35 (56.5%)	0	27 (43.5%)
Q6. Severe hyperkalemia	36 (58.1%)	0	26 (41.9%)
Q7. Posterior MI	46 (74.2%)	3 (4.8%)	13 (21%)
Q8. Pre-excited atrial fibrillation	55 (88.7%)	0	7 (11.3%)
Q9. Dual chamber pacing	19 (30.6%)	31 (50%)	12 (19.4%)
Q10. Tri-fascicular block	51 (82.3%)	0	11 (17.7%)

**Figure 5:**
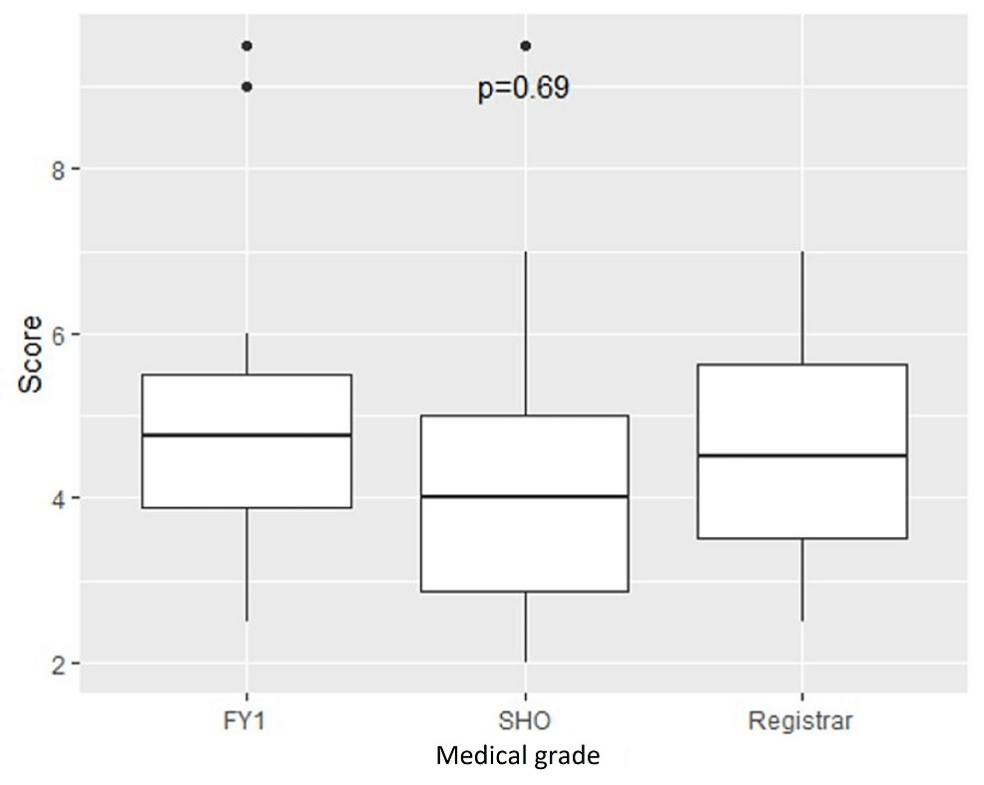
Association of medical grade and score on ECG quiz

No association was found between junior doctor grade and overall score on the ECG assessment, with the mean score percentage among FY1, SHO and registrar level doctors being 50%, 41%, and 46%, respectively (p=0.69) (Figure 5). Years of experience post-graduation similarly had no bearing on quiz scores.

**Figure 6:**
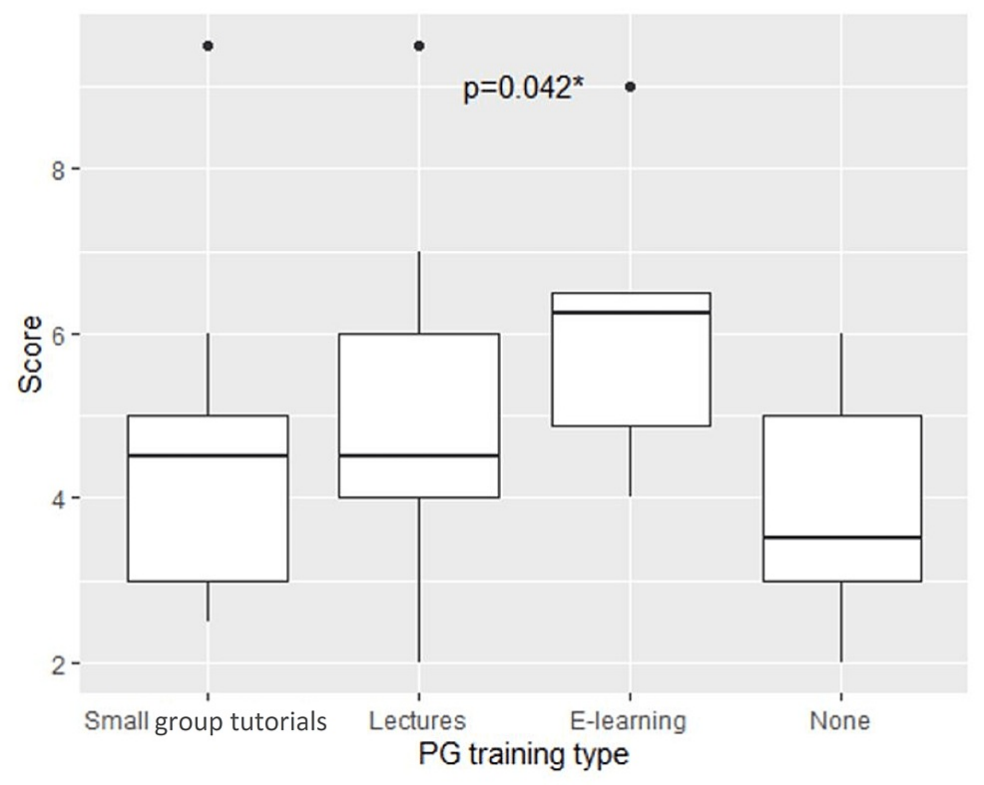
Association between postgraduate teaching method and score on ECG quiz

**Figure 7:**
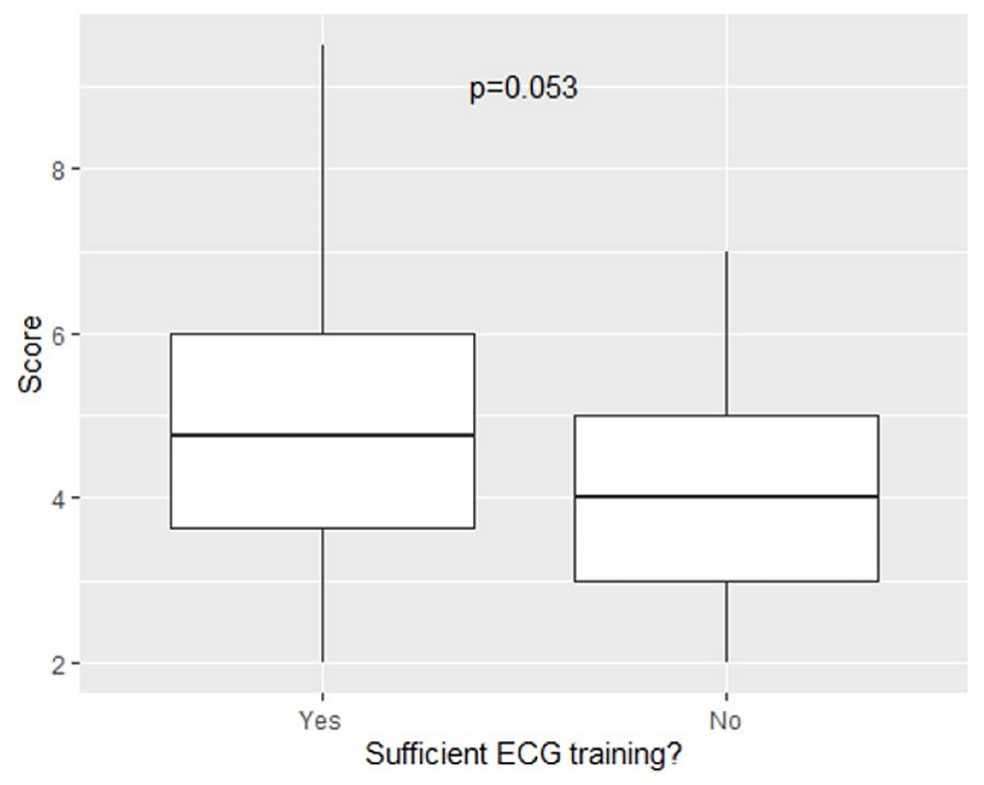
Association believing having had sufficient training and score on ECG quiz

Internet-based modules as postgraduate learning were associated with higher scores on the assessment, reaching statistical significance (p=0.042) (Figure 6). Neither the type of undergraduate ECG teaching nor independent ECG learning method demonstrated an association with interpretation competence; however, those that undertook no additional ECG teaching fared worse than those who did. There was a trend towards participants who felt they had sufficient ECG training to score higher than their counterparts, but this was not statistically significant (p=0.053) as demonstrated in Figure 7.

Participants who felt more confident also showed a trend towards higher scores, as did junior doctors that more frequently had their ECG interpretations checked by another clinician.

Whether a participant had a cardiology rotation or not, approximate number of ECGs interpreted per week or whether they graduated from the UK or abroad demonstrated no association with the overall ECG assessment score.

## Discussion

Electrocardiography is used in the acute clinical setting to diagnose or rule out life-threatening pathologies. ECG interpretation is a basic skill that all junior doctors should have at least a basic degree of proficiency in, as outlined in the Foundation program curriculum [5]. Previous studies have demonstrated varying levels of competence and confidence among doctors and the factors associated with these remain poorly understood. The potential for diagnostic inaccuracies in interpretation leading to delays in investigation, treatment, or discharge stipulates further study in this regard.

Our study in one of the largest acute NHS trusts in the UK, demonstrated generally low levels of competence in ECG interpretation, the mean score on the ECG assessment being 45% in the study group. The level of competence for all those who were eligible to participate is likely to be less as there was probable self-selection bias given participation was voluntary, hence those who felt more confident at ECG interpretation could have been more likely to take part. Free-text answers were the chosen method of recording responses as this was felt to be more closely resemblant to real-world practice as opposed to utilizing multiple choice answers.

There was obvious variation in the ECG teaching approaches experienced by participants at undergraduate and postgraduate levels. The association of internet-based module learning at postgraduate learning level with higher scores on the assessment was likely spurious given the small number of junior doctors that partook in this method (n=9). From the data collected and analyzed, it is not possible to recommend one specific teaching strategy over another and as is the case with most medical education strategies, different teaching strategies will be optimal for different learners, and this is in keeping with previous research where no single method has been established as superior to others [4,6]. Participants who checked their interpretations with another clinician showed a trend toward higher scores, potentially explained by the additional feedback and ad-hoc teaching they received compared to their peers.

Similarly, low levels of ECG interpretation competence among junior doctors and newly graduating medical students were demonstrated in previous studies [2,3,7]. In terms of difficulty with specific ECG patterns, Gillespie et al also showed junior doctors struggled with identifying posterior myocardial infarctions as was evident in our study [2].

In contrast to the findings of this study, seniority of trainees was associated with higher levels of competency in a study conducted on emergency medicine trainees in South Africa [8], another factor previously demonstrated to impact interpretation accuracy was volume of ECGs interpreted per week, with more than 20 ECGs associated with higher levels of competency [9]. This did not impact assessment scores in the current study.

The lack of association between grade of junior doctor (FY1, SHO, and registrar) and ECG interpretation competence is significant to practice as currently at the emergency department, Manchester Royal Infirmary, only “registrars or above” are permitted to interpret and sign that ECGs done at initial triage have been reviewed by a clinician. The competence of those above Registrar level is assumed whereas FY1s and SHOs are required to undertake further learning and pass an assessment within the department to independently “sign off” ECGs. This study did not include any Emergency department personnel; however, based on the results of data analysis, the authors recommend further studies assessing competence among different grades of doctors to substantiate any assumptions on ECG interpretation skills.

A number of limitations existed in our study. The previously described possible self-selection bias could have contributed to higher overall mean scores. Given the time-consuming nature of free-text answers, a compromise between validity and reliability had to be reached and a conscious decision to include less ECGs to maximize response rate but answered by free-text rather than multiple choice questions (MCQs) was made to mimic real-life practice, this low number of questions could have impacted the reliability of scores.

## Conclusions

In conclusion, this study demonstrated low overall levels of competency among junior doctors in a large acute NHS trust regardless of grade. Types of undergraduate, postgraduate, or independent learning did not impact ECG interpretation accuracy; however, there was a trend towards higher levels of competency among those who felt they had undergone sufficient ECG teaching and those who sought regular feedback from other clinicians.
